# Hepatitis C virus transmission among people who inject drugs in rural United States: mathematical modeling study using stochastic agent-based network simulation

**DOI:** 10.1093/aje/kwaf052

**Published:** 2025-07-17

**Authors:** Lin Zhu, Jennifer R Havens, Abby E Rudolph, April M Young, Golnaz Eftekhari Yazdi, William W Thompson, Liesl M Hagan, Liisa M Randall, Jianing Wang, Rebecca Earnest, Shayla Nolen, Benjamin P Linas, Joshua A Salomon

**Affiliations:** Department of Global Health and Population, Harvard T.H. Chan School of Public Health, Boston, MA, United States; Department of Health Policy, School of Medicine, Stanford University, Stanford, CA, United States; Center on Drug and Alcohol Research, University of Kentucky College of Medicine, Lexington, KY, United States; Department of Epidemiology and Biostatistics, Temple University College of Public Health, Philadelphia, PA, United States; Center on Drug and Alcohol Research, University of Kentucky College of Medicine, Lexington, KY, United States; Department of Epidemiology, University of Kentucky College of Public Health, Lexington, KY, United States; Section of Infectious Disease, Department of Medicine, Boston Medical Center, Boston, MA, United States; Prevention Branch, Division of Viral Hepatitis, Centers for Disease Control and Prevention, Atlanta, GA, United States; Prevention Branch, Division of Viral Hepatitis, Centers for Disease Control and Prevention, Atlanta, GA, United States; Bureau of Infectious Disease and Laboratory Sciences, Massachusetts Department of Public Health, Boston, MA, United States; Section of Infectious Disease, Department of Medicine, Boston Medical Center, Boston, MA, United States; Department of Global Health and Population, Harvard T.H. Chan School of Public Health, Boston, MA, United States; Section of Infectious Disease, Department of Medicine, Boston Medical Center, Boston, MA, United States; Section of Infectious Disease, Department of Medicine, Boston Medical Center, Boston, MA, United States; Department of Epidemiology, Boston University School of Public Health, Boston, MA, United States; Department of Health Policy, School of Medicine, Stanford University, Stanford, CA, United States

**Keywords:** people who inject drugs, hepatitis C, social network, simulation model, transmission, rural population, heterogeneity

## Abstract

People who inject drugs (PWID) account for the majority of hepatitis C virus (HCV) infections in the United States. The injection-equipment-sharing network likely plays an important role in shaping the dynamics of HCV transmission. Recognizing the emerging HCV epidemic in rural communities, we developed an agent-based network simulation model of HCV transmission via injection equipment sharing and used data on rural PWID networks to inform model parameterization and calibration. We then simulated an array of networks that varied key network properties to understand their impact on the magnitude and distribution of HCV incidence. The results show substantial heterogeneity in HCV acquisition risks across the network, summarized using the Gini coefficient. In addition, although PWID with fewer injection partners had lower incidence, they collectively acquired more infections due to their larger population size. Higher prevalence, average number of partners, and homophily in HCV infection were associated with lower heterogeneity in infection risk across the network and higher overall incidence; other network properties including population size did not have a substantial impact. Our findings illustrate the heterogeneity of HCV transmission among PWID and suggest key network properties that could be measured, evaluated, or considered in the design of interventions for PWID in future studies.

## Introduction

Approximately 2.4 million adults in the United States are estimated to have hepatitis C virus (HCV) infection,^1^ and the number of deaths attributable to hepatitis C was approximately 14 000 in 2021.^2^ Injection drug use has been the dominant route of HCV transmission in the United States over the past 3 decades,^3^^,^^4^ and people who inject drugs (PWID) account for an estimated 67% of new HCV infections.^5^ Fueled by the ongoing opioid crisis, incidence of HCV infection steadily increased from 2013 to 2021.^6^ Rural areas have experienced emerging epidemics of HCV infection related to injection drug use, but research in rural areas is limited.^7^ Although the total numbers of acute and chronic HCV infections are higher in urban areas than in rural areas, the rates are both higher in rural areas.^8^^,^^9^

Interventions to reduce drug use and harm reduction programs are important elements in the prevention and control of HCV transmission.^10^ The advent of direct-acting antiviral (DAA) treatment for HCV infection has raised prospects for eliminating hepatitis C, as the treatments are highly effective, have minimal side effects, and require only 8-12 weeks of oral medication,^11^^,^^12^ but they are costly.^13^ Understanding the patterns of HCV transmission among PWID may help to optimize intervention strategies for the prevention and control of HCV infection.

Mathematical models are often used to simulate HCV transmission and control.^14^^-^^17^ Hepatitis C virus is transmitted through the sharing of injection equipment (needles, syringes, and other equipment used to prepare and consume drugs) among PWID. Studies have shown that the properties of transmission networks can play an important role in disease transmission dynamics.^18^^-^^24^ For example, PWID network modeling studies found that the impact of hepatitis C “treatment as prevention” strategy varies depending on the structure of the injection network.^23^^,^^25^ Consequently, network properties may be important to consider when understanding HCV transmission dynamics and designing hepatitis C elimination strategies.^26^

Models incorporating injection networks have been used to understand HCV transmission and evaluate the potential impact of different treatment strategies.^27^^-^^36^ The majority of these models have inferred network properties from data collected through chain-referral sampling, including snowball sampling^28^^-^^31^ and respondent-driven sampling (RDS).^32^ Typically, analyses have simulated transmission using either a replicated sample network^29^ or a network that was reconstructed with statistical models of sample estimates.^28^^,^^30^^-^^32^ We have demonstrated previously that when employing RDS, the estimates of network properties from the sampled network may differ from those for the underlying network.^37^ In addition, PWID network properties (eg, average number of partners per person) and hepatitis C prevalence can vary by location and over time. To improve understanding of the generalizability of findings across different networks, the aim of this study was to examine how differences in network properties and hepatitis C prevalence may impact the overall incidence of HCV infection in a PWID population and the distribution of incident infections among individuals in the population.

## Methods

### Analytic overview

We combined stochastic simulation of injection-equipment-sharing networks among PWID with a simple model of HCV transmission to consider how network properties can shape transmission dynamics. We analyzed data from an empirical study of people who use drugs (PWUD) in a rural setting (Central Appalachia) and reviewed published literature on rural PWID networks in the United States to generate a range of representative parameters and to calibrate the model. We then simulated an array of distinct networks that varied in terms of key network and epidemiologic parameters to explore their impact on incidence of HCV infection and heterogeneity of HCV infection risk among PWID.

### Data

To inform the simulation of the injection network and HCV transmission in the base-case scenario, we analyzed data from the Social Networks among Appalachian People (SNAP) study, which is a longitudinal study among a cohort of PWUD in rural Eastern Kentucky. This area and the study sample are characterized by a high burden of opioid-related overdose and HCV infection.^38^^,^^39^ The SNAP study recruitment methods and network data have been described in detail previously.^40^^-^^46^ In brief, 503 PWUD were recruited through RDS between 2008 and 2010 and followed up at 6-month intervals. Information collected through interviewer-administered questionnaires included demographics, injection drug use behaviors, and HCV antibody testing results. Participants also reported up to 24 people with whom they used drugs, had sex, or received social support in the past 6 months. Participants were asked whether they injected drugs with each of the reported individuals. Fuzzy look-up was used to cross-reference the names of reported alters with those of participants to produce possible matches based on name similarity.^42^ We used baseline and 2 years’ follow-up data from 287 SNAP participants who reported injecting drugs during the 6 months prior to recruitment, referred to hereafter as the “SNAP PWID sample.” We defined the partnership as an injection-equipment-sharing partnership if at least 1 member of the pair reported sharing needles or cookers with the other. We did not distinguish between needle sharing and cooker sharing or the direction of sharing, because all injection equipment is a potential vector for HCV transmission and can be used repeatedly.^47^  Table 1 summarizes the demographics, HCV serostatus, and network statistics of the observed injection-equipment-sharing network among the SNAP PWID sample.

**Table 1:** Demographics, HCV infection, and network statistics of 287 PWID in SNAP^a^ study, 2008-2010.

**Characteristics**	***N* (%)**
Age (yr), mean (SD)	31.6 (7.9)
Male	169 (59)
Non-Hispanic White	269 (94)
HCV antibody positive^b^ at baseline	168 (59)
HCV seroconversion^b^ incidence rate, per 1000 person-years-at-risk	248
Isolates^c^	105 (37)
Overall mean degree^d^	1.43
Ratio of mean degree between HCV antibody positive and antibody negative^b^ PWID	1.73
HCV serodiscordant^b^ partnerships^e^	37 (29)
Partnership^e^ triangles that are closed^f^	13 (7)

Abbreviations: HCV, hepatitis C virus; PWID, people who inject drugs; SNAP, Social Networks among Appalachian People.

a The SNAP study is a longitudinal study of a cohort of people who use drugs in rural Eastern Kentucky.

b HCV antibody positive reflects current or past infection, where the infected individual has seroconverted (ie, developed antibody).

c PWID that do not share injection equipment with others.

d Average number of injection-equipment-sharing partners per person.

e The partnership is defined as an injection-equipment-sharing partnership if at least 1 member of the pair reported sharing needles or cookers with the other, including those to a partner outside the 287 PWID sample. To calculate HCV serodiscordant partnerships, we only used those between the 287 PWID sample since the serostatus of people outside the sample were unknown.

f *N* depicts the number of triangles (3 PWID sharing injection equipment with each other), and % is *N* divided by the number of 2-stars (ie, 2 PWID sharing with another common PWID; the value is 197).

### Literature review of rural PWID networks

To account for potential variations in injection networks and investigate the impact of network properties on HCV transmission patterns, we simulated 12 distinct network variants in which we varied 1 parameter from the base-case network at a time. We defined the range for each parameter based on values reported in the published literature on rural PWID in the United States (Table 2). While the results from each analysis were variable in terms of how partners were defined, we selected a range of parameters to be inclusive and feasible. When not available, we varied the parameter values to include a feasible range spanning values lower and higher than the value observed in the SNAP PWID sample.

**Table 2:** Rural people who inject drugs (PWID) characteristics based on a literature review of rural PWID networks and hepatitis C prevalence in the United States^a^.

**Location**	**Study year(s)**	**Sample size**	**Definition of partner**	**Time counted**	**Mean degree** ^**b**^	**HCV seroprevalence** ^**c**^	**Reference**
Puerto Rico	2015	315	Current injection partners	Past 12 months	8 (median)	78%^d^	^48^
			Use a needle or cooker after someone else injected with it		1.2 for needle, 4.5 for cooker		^49^
			Use same cooker, cotton, or water that a partner had already used		4.9 among HCV antibody positive; 3.0 among HCV antibody negative		^50^
			Used the syringe after their partner did		0.857		^51^
Indiana	2015	411	Needle sharing	Past 12 months	2.37	NA	^52^
New York (young PWID)	2012	115	Two inconsistent definitions: “Inject with” in the table, “needle sharing” in the text	Not defined	Overall, 55.7% have a degree not greater than 1, 46.7% of those who were HCV antibody positive and 59.4% of those who were HCV antibody negative had a degree not greater than 1	34%^d^	^53^
Connecticut	2008-2012	462	Not defined	Past 6 months	53.4% have equal to or greater than 6 injection partners	39.20%	^54^
Wisconsin	2010-2012	243	Share needle, cottons, water, or “parallel” (a term developed to describe individuals who ;used injection drugs in the same vicinity as a named partner, but unsure whether needles or other equipment were shared)	Not defined	3.9	69.60%^e^	^55^

Abbreviations: HCV, hepatitis C virus; PWID, people who inject drugs; SNAP, Social Networks among Appalachian People.

a Our literature search queried the PubMed database using the search terms (“people who inject drugs” OR “persons who inject drugs” OR “injection drug users” OR “injecting drug users” OR “PWID” OR “IDU”) AND (“network*” OR “partnership” OR “sharing” OR “homophily” OR “mean degree” OR “transitivity” OR “needle*” OR “cooker*” OR “works” OR “syringe*”) AND “rural,” augmented by additional citations from these papers. We excluded results from the SNAP study, a longitudinal study of a cohort of people who use drugs in rural Eastern Kentucky, which we used for model parameterization and calibration in the main analysis.

b Average number of injection-equipment-sharing partners per person.

c HCV seroprevalence indicates HCV antibody positive/reactive, and a reactive HCV antibody test represents current infection or past cleared infection.

d Measured by rapid antibody testing.

e Mentioned as HCV testing status of positive or negative; no testing method was provided and assumed to be HCV antibody testing.

### Modeling the network

In each simulation, we generated a rural PWID population and assigned baseline HCV antibody status (antibody positivity reflects current or past infection in which the individual has developed HCV antibody/seroconverted). Hepatitis C virus treatment was limited during the period over which the baseline SNAP data were collected,^10^ and the SNAP study and a systematic review and meta-analysis reported an HCV spontaneous clearance rate of approximately 25% among PWID, we therefore assigned 25% of the antibody-positive PWID to have no current infection and noninfectious.^56–58^

**Figure 1:**
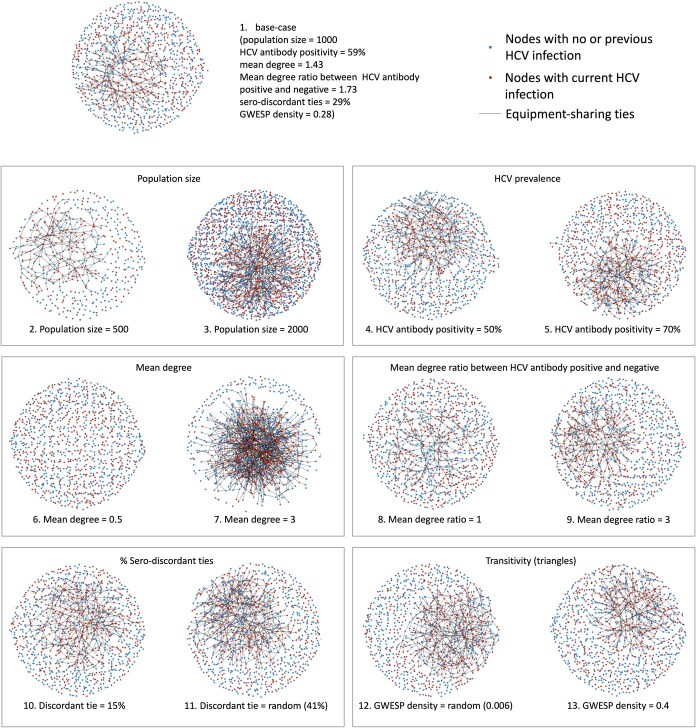
Network plots of the baseline network and 12 network variants. The numbering of the subfigures corresponds to the network ID in Table 3, followed by a brief description of the parameter that is varied from the base-case network. In each plot, the nodes represent people who inject drugs (PWID) individuals with different hepatitis C virus (HCV) infection status. The ties between them represent the injection-equipment-sharing partnerships.

We then used exponential random graph models (ERGMs) operationalized with the Statnet package (R package, version 2019.6)^59^^,^^60^ to simulate injection-equipment-sharing partnerships that define the network for each simulated population. An ERGM predicts the probability that any 2 individuals (nodes) in the population share injection equipment (have a tie). The probability of a tie is determined based on attributes specific to the individual (nodal level), the partnership between 2 individuals (dyadic level), and/or the whole network (network level).^59^ We included ERGM terms in our models to represent the following network statistics: (1) mean degree, that is, the average number of ties per node^61^; (2) mean degree by HCV antibody status, summarized as a mean degree ratio between people who are HCV antibody positive and negative; (3) proportion of isolates, ie, individuals with no ties; (4) homophily in infection status, summarized as the proportions of ties between those who have the same HCV antibody positivity; and (5) transitivity, which is the tendency for individuals to share injection equipment with the partners of their injection partners, that is, forming triangles.^61^ A network statistic known as “geometrically weighted edgewise shared partner” (GWESP) is commonly used to measure transitivity in ERGMs.^62^^,^^63^ In the Statnet environment, “GWESP(decay = 0)” counts the number of ties in at least 1 triangle, which we translated into a measure of GWESP density (proportion of ties that are in at least 1 triangle) to facilitate comparison across networks of different sizes or mean degrees. As described above, the range of network statistics used in ERGMs were informed by SNAP network analyses and the literature, which are summarized in Table 3 and depicted visually in Figure 1. Further details of the target network statistics we used to estimate ERGM coefficients are summarized in Table S1.

**Table 3:** Parameters and network statistics for simulations of the network variants.

**Network ID**	**Brief description of the variant**	**Network size**	**Percentage of PWID that is HCV antibody positive (%)**	**Mean degree**	**Mean degree ratio (HCV antibody positive/HCV antibody negative)**	**Percentage of ties with different HCV antibody status (%)**	**Transitivity (GWESP density** ^**a**^ **)**	**Percentage of the samples that are isolates (%)**
1	Base-case^b^	1000	59	1.43	1.73	29	0.28	37
2	Smaller network size	500	59	1.43	1.73	29	0.28	37
3	Larger network size	2000	59	1.43	1.73	29	0.28	37
4	Lower prevalence	1000	50	1.43	1.73	29	0.28	37
5	Higher prevalence	1000	70	1.43	1.73	29	0.28	37
6	Lower mean degree	1000	59	0.5	1.73	29	0.28	Random (66)^c^
7	Higher mean degree	1000	59	3	1.73	29	0.28	Random (9)^c^
8	Lower mean degree ratio	1000	59	1.43	1	29	0.28	37
9	Higher mean degree ratio	1000	59	1.43	3	29	0.28	37
10	Lower % discordant tie	1000	59	1.43	1.73	15	0.28	37
11	Higher % discordant tie	1000	59	1.43	1.73	Random (41)^d^	0.28	37
12	Lower transitivity	1000	59	1.43	1.73	29	Random (0.006)^d^	37
13	Higher transitivity	1000	59	1.43	1.73	29	0.4	37

Abbreviations: ERGM, exponential random graph model; GWESP, geometrically weighted edgewise shared partner; HCV, hepatitis C virus; PWID, people who inject drugs; SNAP, Social Networks among Appalachian People.

a Proportion of ties that are in at least 1 triangle.

b The network statistics and hepatitis C prevalence of the simulated base-case networks were derived from the SNAP PWID sample as shown in Table 1.

c For these 2 network variants with different mean degrees, we did not specify percentage of isolates in the ERGM since percentage of isolates in a network is probably correlated with mean degree. The average percentage of isolates randomly emerged in the simulated networks was 66% for the lower mean degree network and 9% for the higher mean degree network.

d For these network variants, we did not find information from the literature to specify the bounds. When there is homophily in infection status (ie, preference for ties between those with the same HCV status), the proportion of ties between those with discordant HCV status will be lower. For the high homophily in infection status scenario, we used half of the base-case value as the lower bound. We did not observe a preference for partnerships between those with different HCV antibody status among PWID, so we used random mixing (ie, excluding this term in ERGM, letting discordant ties to emerge at random) as the upper bound, and the average percentage of discordant partnerships in simulated networks with this setting was 41%. Similarly, we used random emergence as the lower bound for transitivity (GWESP density), and the mean in the simulated networks was 0.006.

e In addition to the network parameters in the table, we used a calibrated monthly transmission probability of 0.031 for all serodiscordant ties, and used an HCV spontaneous clearance rate of 25%, as described in the method section.

### Simulation and calibration of HCV transmission

Only individuals that have current infection can transmit HCV. We simulated transmission in monthly time steps. At each time step, the model evaluates each tie in a random order. If the HCV infection status of the 2 nodes of a tie is discordant, HCV transmission can occur based on a monthly transmission probability. During the first 6 months after acute infection, individuals have a cumulative spontaneous clearance probability of 25%.^58^ While we did not explicitly simulate HCV treatment or interventions, such as syringe services programs, for this study, which were both uncommon during the period over which SNAP data were collected,^10^^,^^64^ the prevalence and incidence of HCV infection and reported drug use behaviors among the SNAP PWID sample will implicitly reflect current levels of treatment and other interventions. As such, changes in intervention coverage can be represented by alterations to the network properties in the 12 network variants. For instance, if there is coverage of syringe services, individuals are likely to have fewer injection-equipment-sharing partners, resulting in a lower mean degree, as examined in network variant 6. Similarly, if HCV treatment is available, individuals can be cured, leading to a reduction in the number of infected individuals, and subsequently lower prevalence, as examined in network variant 4. Given that we employed a simulation time horizon of 1 year, lower prevalence should yield effects similar to those of ongoing treatment coverage. Furthermore, the availability of HCV treatments and harm reduction programs can vary substantially across different locations. Therefore, we demonstrate how these changes, as reflected in network properties, can impact transmission dynamics, as shown in the network variants examined.

The monthly transmission probability per HCV-discordant tie was calibrated to match the seroconversion incidence rate estimated using the SNAP follow-up data: using the base-case network, we first ran simulations with a series of transmission probabilities between 0 and 1 and compared the simulated seroconversion incidences to the estimated incidence in SNAP. To generate a distribution of incidence rates that include the SNAP estimate, the range of transmission probabilities for serodiscordant pairs was determined to be between 0.03 and 0.06 per month. We then used a Bayesian approach for model calibration,^65^ in which we ran 10 000 simulations with transmission probabilities sampled from a uniform distribution across that range and calculated the likelihood for each run with a Poisson distribution. We finally calculated the mean of the 10 000 transmission probabilities weighted by their likelihood, which equals 0.031 per discordant tie per month. We fixed this parameter for simulations across all network variants. Because the primary aim in this study was to identify the effects of network properties on the distribution of risk across network members, we did not incorporate additional sources of heterogeneity in transmission probability across serodiscordant pairs.

### Analyses

To examine the distribution of infection risk across individuals in each simulated network, we plotted Lorenz curves of the risk of incident infection among all susceptible individuals in the simulated networks. A Lorenz curve summarizes the concentration of risk in a given population by first ordering individuals in that population by increasing risks and then plotting the cumulative share of risk against the cumulative share of population.^66^ To do so, we first used ERGMs to generate 100 stochastic baseline networks for each network variant. For each baseline network, we ran 100 stochastic iterations of the HCV transmission simulation. For each susceptible individual in the baseline network, we recorded the percentage of the 100 iterations in which the individual was infected by the end of a 1-year period to represent the individual’s risk of infection. We ordered the individuals by their risks for each baseline network and averaged the results across the 100 stochastic baseline networks to plot the Lorenz curves for each network variant. We also calculated the Gini coefficient associated with each Lorenz curve, which is the area between the diagonal line (equal distribution of risk) and the curve divided by the total area under the diagonal line. The values are expressed on a scale between 0 and 1, in which 0 is perfect equality, and higher numbers indicate more heterogeneity.^66^ To understand how an individual’s degree impacts infection risk, we compared the total number of infections and incidence rate between different degree groups for each network variant.

## Results

**Figure 2:**
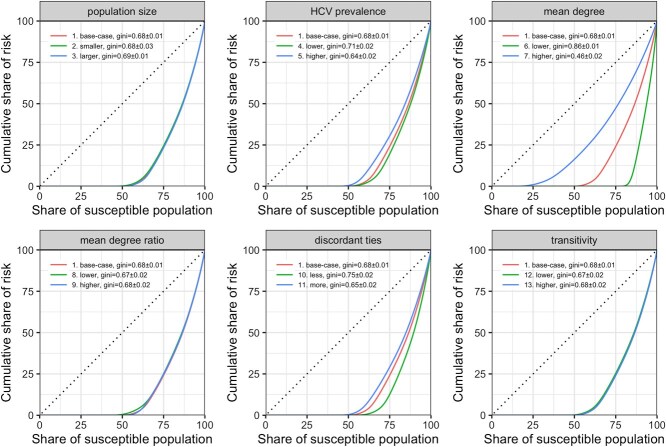
Lorenz curves (cumulative risk of hepatitis C virus [HCV] infection per share of population) for the base-case network and the 12 network variants. Each panel shows the Lorenz curves of the base-case network and 2 network variants that have 1 network-generating parameter (panel title) varied from the base-case. In each panel, the results of each network variant are depicted in different colors, and the legend specifies the network ID corresponding to those in Table 3 and Figure 1. The Lorenz curves show the cumulative share of risk (y-axis) per cumulative share of susceptible population (x-axis). The further the Lorenz curve is from the diagonal line (perfect equality of risk), the more heterogeneous the infection risks are among the individuals, which is also quantified by the Gini coefficient (the area between the diagonal line and the curve divided by the total area under the diagonal line), which has a range between 0 (perfectly homogeneous) and 1 (perfectly heterogeneous).

Figure 2 shows Lorenz curves for all network variants, with associated Gini coefficients ranging from 0.46 (higher mean degree) to 0.86 (lower mean degree), indicating heterogeneous risks of HCV acquisition across susceptible individuals in each network even though the same monthly transmission probability was used between all discordant partnerships. In the base-case network, for example, approximately 50% of the susceptible individuals did not seroconvert (ie, infection risk = 0), individuals with the highest risk (top 25%) contributed to approximately 75% of total infections, and the Gini coefficient was 0.68.

Parameters including higher baseline hepatitis C prevalence, higher mean degree, and higher percentage of serodiscordant ties (less homophily in infection status) were associated with less heterogeneity in risk, while variations in population size, mean degree ratio between seropositive and seronegative individuals, and transitivity did not substantially alter the extent of risk heterogeneity from that of the base-case network.

**Figure 3:**
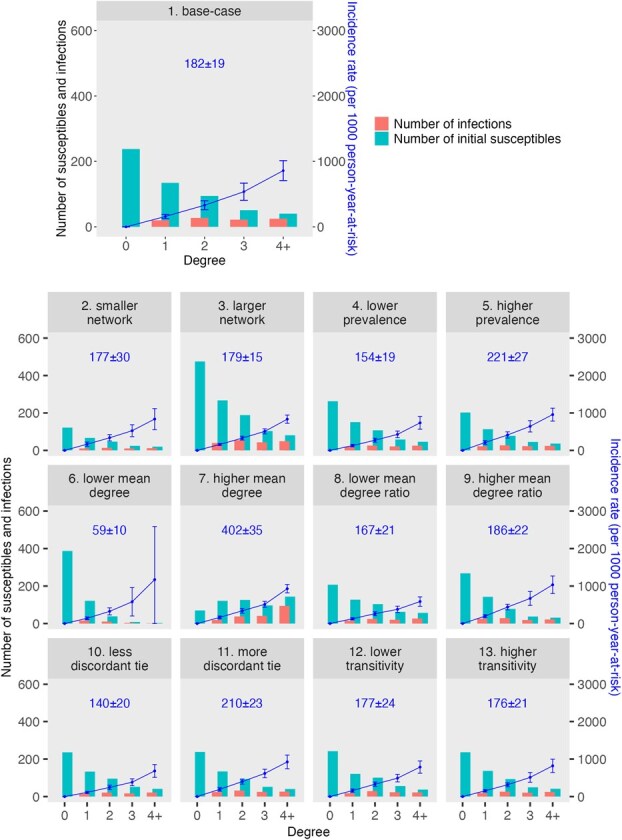
Degree distribution, number of hepatitis C virus (HCV) infections, and incidence rate among susceptible people who inject drugs (PWID). Each panel shows results from 1 network variant as described in the panel title, and the network ID corresponds to those in Table 3 and Figure 1. In each panel, the x-axis shows different degree groups, and the y-axis on the left corresponds to the bars, which show the number of susceptible PWID at the start of simulation and the number of infections over a 1-year time horizon in each degree group. The y-axis on the right corresponds to the points and error bars (SD), which show the incidence rate in each degree group. The number annotated in the upper middle of each panel shows the mean ± SD of the overall incidence rate.

Figure 3 shows the degree distribution, number of infections, and incidence rate in each degree group among susceptible PWID in each network variant. Incidence rates increased with increasing degree in all network variants. Although the incidence rate among those with larger degrees (more injection partners) was higher, they comprised a small fraction of the simulated population. Thus, most infections occurred among those with smaller degrees (fewer injection partners) who comprised a larger fraction of the simulated population. For example, in the base-case network, there were an average of 558 susceptible individuals at the start of simulation, 238 (43%) of whom did not have any injection partners, and the total number of infections was 94, corresponding to an incidence rate of 182 per 1000 person-years-at-risk. The incidence rate among those with a degree of at least 4 was the highest (858 per 1000 person-years-at-risk). Seven percent of susceptible individuals had at least 4 injection partners, and they accounted for 26% of all infections. On the other hand, the incidence rate among those with a degree of 2 (ie, 2 injection partners) was 330 per 1000 person-years-at-risk. Given that 17% of the susceptible individuals in this simulated network had a degree of 2, they accounted for 29% of all infections. Of the 94 total infections, 74% occurred in individuals with a degree of 1-3 (ie, 1-3 injection partners).

The overall incidence rate increased as the average number of injection partners, hepatitis C prevalence at baseline, or HCV serodiscordant ties increased. For example, the overall incidence was over 2 times as high in the network variants with higher mean degree (ie, more partners per person on average). The incidence was more than 1 SD higher in network variants with a higher baseline hepatitis C prevalence or more HCV serodiscordant ties. The incidence was less impacted by changes in the population size, mean degree ratio between seropositive and seronegative individuals, or transitivity.

## Discussion

In this article, we examine how properties of the injection-equipment-sharing network impact HCV transmission dynamics among PWID using an agent-based network model. Intuitively, the overall incidence in the PWID network increased when baseline prevalence was higher, when individuals had more injection partners on average, or when there were more serodiscordant partnerships.

In our simulation results, the risk of acquiring HCV infection varied across individuals, with the highest risk of infection occurring among a small proportion of individuals—those with more injection partners. This finding was consistent across all the simulated network variants (Figures 2 and 3). By design, we did not incorporate different HCV transmission risks in relation to individuals’ injection frequency or type of equipment shared (ie, syringes vs cookers) but rather used the same HCV transmission probability for each HCV-discordant tie. This design choice was made in accordance with our main aim in this article to characterize the dispersion of infection risk attributable to network properties rather than to additional individual factors.

To explore the impact of an individual’s degree on HCV infection incidence, we calculated incidence rates stratified by degree (Figure 3). Among susceptible individuals, those with more injection partners were more likely to be infected in the simulation. However, because most individuals had fewer than 3 partners, most of the infections occurred among individuals with fewer partners. It is important to note that our analysis focused on the acquisition of infection by degree groups, rather than onward transmission, a distinction that should be taken into account when designing intervention programs. In another study, we compared the impact of degree-based allocation with random allocation for curative (eg, DAA treatment) and preventive (eg, harm-reduction, such as syringe services) interventions, and found that prioritizing higher-degree individuals for preventive interventions while employing random allocation for curative interventions resulted in greater reductions in the overall prevalence and incidence of HCV infection.^67^ This finding indicates that higher-degree individuals may have contributed to more onward transmission. Nevertheless, both studies demonstrate the importance of treating all individuals, including those with a lower degree and risk of infection.

Although an individual’s degree may play an important role in selecting an allocation approach for preventive interventions, the effort that should be devoted to identifying high-degree individuals can vary between networks with different magnitude of heterogeneity in HCV infection risks. For example, if heterogeneity in a population is substantially higher than average, greater effort should be placed on identifying high-risk individuals. On the contrary, if risk is evenly diffused across the network, increasing intervention coverage in the population may have the highest priority. Our findings (Figure 2) show that infection risks were less heterogeneous when the baseline hepatitis C prevalence was high, individuals had on average more injection partners, or a greater proportion of injection partners were serodiscordant, which are properties to consider when determining intervention emphasis for a population. Of note, we did not fix the percentage of isolates to be equal to the base-case network in the 2 network variants that altered the mean degree (Table 3) based on the assumption that mean degree and percentage of isolates may be correlated (eg, network with higher mean degree may have less isolates). If the percentage of isolates was fixed, we expect the impact of mean degree on risk heterogeneity to be less substantial.

Empirical studies on network properties of PWID are sparse, and the few studies described in Table 2 often reported a limited number of network properties. Most PWID network models assume an artificial population size because the underlying population size is often unknown due in part to injection drug use being stigmatized and illegal.^23^^,^^32^ Our findings suggest that network properties including population size, relative difference in the number of injection partners between those who are seropositive vs negative, and transitivity of partnerships did not contribute to the heterogeneity of infection risk at the individual level or overall incidence at the population level, which could increase confidence in results from simulation studies that use an artificial population size and increase the utility of datasets that have limited information on network properties. However, our findings do emphasize the importance of considering infection prevalence, mean degree (average number of partners), and homophily in infection status in PWID network studies. As social, cultural, and other factors may influence PWID network properties, we need to take these variations into account when designing elimination strategies.

We modeled rural PWID in this study based on the SNAP data and literature review on rural PWID networks. However, population demographics, epidemiology of HCV infection, and network properties can vary greatly across different PWID populations. We hence focus on which and how network properties affect HCV transmission instead of providing exact implications for any specific PWID population. The coverage of interventions, such as HCV treatment and harm reduction programs, can also vary across locations and over time. As explained in the methods section, these interventions can be implicitly reflected by the changes in prevalence and network properties such as mean degree, and we demonstrate their impact using different network variants.

Our model has several limitations. First, we developed a static network model because the mean partnership duration in the SNAP PWID sample and in another urban PWID sample was 10 years.^32^^,^^46^ However, formation and dissolution of partnerships, and population dynamics, such as initiation, cessation, and relapse of injection drug, use may affect disease transmission dynamics.^45^ Given our use of a static model, we only examined transmission over a 1-year period. Future model extensions could include addition of network dynamics. Second, we only modeled and evaluated the impact of a limited number of network statistics. Other network statistics, such as k-core (maximal connected subgroup in which all nodes have a degree of at least k) membership, clique membership, and average path length (average number of ties along the shortest path between all possible pairs of individuals), may also impact HCV transmission and require future consideration. Third, we opted to use a single transmission probability across all discordant relationships; however, different transmission probabilities based on frequency of injection equipment sharing, type of equipment shared, and direction of equipment sharing can further increase the heterogeneity of the transmission risk or influence the impact of an individual’s network position on their infection risk. Incorporating this heterogeneity, which we considered to be outside the scope of the present study, would require comprehensive data on interactions between these factors and network position, as well as understanding how each factor and their interactions influence transmission risk, for example, the relative risk of transmission from needle sharing compared to cooker sharing. Future studies could incorporate more individual factors and heterogeneity to explore these interaction effects as additional data become available. In addition, we fixed the calibrated monthly transmission probability per discordant partnership in simulations across all network variants to investigate the potential impact of each network property on HCV transmission dynamics. If we recalibrate this parameter for each network variant utilizing the seroconversion incidence estimated from the SNAP data, all network variants would produce the same overall incidence. However, we do not anticipate that this recalibration would substantially influence the impact of network properties on the heterogeneity of HCV transmission.

## Conclusion

We developed a stochastic agent-based network model of injection-related HCV transmission among PWID that was fitted to parameters obtained from a longitudinal cohort of rural PWID in the United States and literature on rural PWID networks and highlight the impact of varying network properties on the heterogeneity in HCV acquisition risk across susceptible individuals in the population and the overall incidence of HCV infection in the population. In particular, among susceptible individuals, the risk of HCV acquisition increased with the number of injection partners; however, those with fewer partners comprised the majority of the population and therefore the majority of incident cases. This suggests that while it may be more efficient to target prevention interventions to those with more injection partners when resources are limited, it is important to cover all PWID at risk to achieve hepatitis C elimination. Second, heterogeneity in HCV infection risks was reduced in contexts where HCV transmission is more likely: higher baseline prevalence of hepatitis C, susceptible individuals have on average more injection partners, or a greater proportion of injection partners are serodiscordant. And variation in the heterogeneity magnitude in different populations may inform the focus of public health intervention strategies. Although an individual’s degree may play an important role in selecting an allocation approach for interventions, in a population with a substantially higher-than-average heterogeneity, greater effort should be devoted to identifying high-risk individuals, while in a population with evenly diffused risks, the priority should be on increasing intervention coverage in the population.

## Supplementary Material

Web_Material_kwaf052
