# Supplementary material for: Hepatitis C virus transmission among people who inject drugs in rural United States: mathematical modeling study using stochastic agent-based network simulation
Source: Am J Epidemiol. 2025 Jul 17;195(4):937–47. doi: 10.1093/aje/kwaf052 (PMC13066326; doi:10.1093/aje/kwaf052)
Supplement: Web_Material_kwaf052 [file web_material_kwaf052.zip › supplement.docx]

**Hepatitis C virus transmission among people who inject drugs in rural United States: mathematical modeling study using stochastic agent-based network simulation**

**Supplement**

Lin Zhu, Jennifer R. Havens, Abby E. Rudolph, April M. Young, Golnaz Eftekhari Yazdi, William W. Thompson, Liesl M. Hagan, Liisa M. Randall, Jianing Wang, Rebecca Earnest, Shayla Nolen, Benjamin P. Linas, Joshua A. Salomon

**Table S1** Target network statistics for the estimation of ERGMs in all network scenarios

| Network ID | edges | gwesp | nodefactor("antibody") | nodemix("antibody") | | | degree(0) |
| --- | --- | --- | --- | --- | --- | --- | --- |
| 1 | 715 | 200 | 1020 | 101 | 207 | 406 | 370 |
| 2 | 358 | 100 | 510 | 51 | 104 | 203 | 185 |
| 3 | 1430 | 400 | 2040 | 202 | 415 | 813 | 740 |
| 4 | 715 | 200 | 906 | 158 | 207 | 349 | 370 |
| 5 | 715 | 200 | 1146 | 38 | 207 | 469 | 370 |
| 6 | 250 | 70 | 357 | 35 | 73 | 142 | NA |
| 7 | 1500 | 420 | 2140 | 212 | 435 | 853 | NA |
| 8 | 715 | 200 | 844 | 189 | 207 | 318 | 370 |
| 9 | 715 | 200 | 1161 | 31 | 207 | 477 | 370 |
| 10 | 715 | 200 | 1020 | 151 | 107 | 456 | 370 |
| 11 | 715 | 200 | 1020 | NA | NA | NA | 370 |
| 12 | 715 | NA | 1020 | 101 | 207 | 406 | 370 |
| 13 | 715 | 286 | 1020 | 101 | 207 | 406 | 370 |
